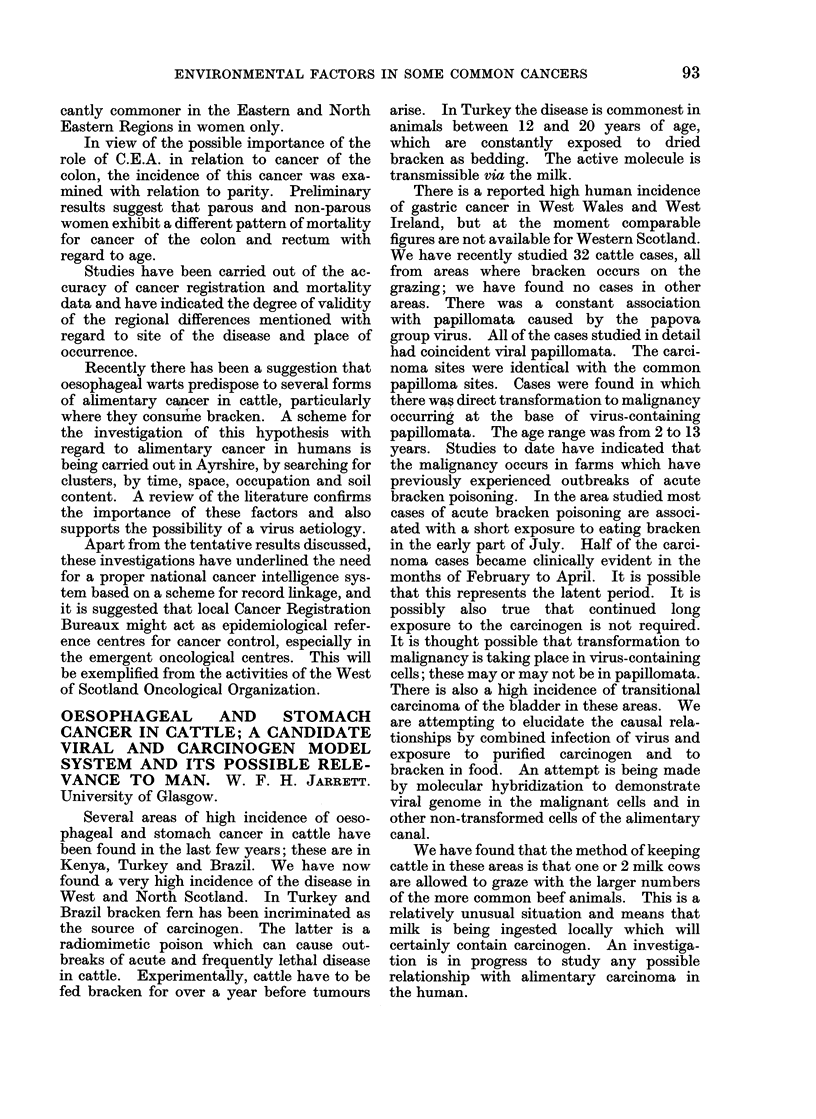# Oesophageal and stomach cancer in cattle; a candidate viral and carcinogen model system and its possible relevance to man.

**DOI:** 10.1038/bjc.1973.121

**Published:** 1973-07

**Authors:** W. F. Jarrett


**OESOPHAGEAL AND STOMACH CANCER IN CATTLE; A CANDIDATE VIRAL AND CARCINOGEN MODEL SYSTEM AND ITS POSSIBLE RELEVANCE TO MAN.** W. F. H. JARRETT. University of Glasgow.

Several areas of high incidence of oesophageal and stomach cancer in cattle have been found in the last few years; these are in Kenya, Turkey and Brazil. We have now found a very high incidence of the disease in West and North Scotland. In Turkey and Brazil bracken fern has been incriminated as the source of carcinogen. The latter is a radiomimetic poison which can cause outbreaks of acute and frequently lethal disease in cattle. Experimentally, cattle have to be fed bracken for over a year before tumours arise. In Turkey the disease is commonest in animals between 12 and 20 years of age, which are constantly exposed to dried bracken as bedding. The active molecule is transmissible *via* the milk.

There is a reported high human incidence of gastric cancer in West Wales and West Ireland, but at the moment comparable figures are not available for Western Scotland. We have recently studied 32 cattle cases, all from areas where bracken occurs on the grazing; we have found no cases in other areas. There was a constant association with papillomata caused by the papova group virus. All of the cases studied in detail had coincident viral papillomata. The carcinoma sites were identical with the common papilloma sites. Cases were found in which there was direct transformation to malignancy occurring at the base of virus-containing papillomata. The age range was from 2 to 13 years. Studies to date have indicated that the malignancy occurs in farms which have previously experienced outbreaks of acute bracken poisoning. In the area studied most cases of acute bracken poisoning are associated with a short exposure to eating bracken in the early part of July. Half of the carcinoma cases became clinically evident in the months of February to April. It is possible that this represents the latent period. It is possibly also true that continued long exposure to the carcinogen is not required. It is thought possible that transformation to malignancy is taking place in virus-containing cells; these may or may not be in papillomata. There is also a high incidence of transitional carcinoma of the bladder in these areas. We are attempting to elucidate the causal relationships by combined infection of virus and exposure to purified carcinogen and to bracken in food. An attempt is being made by molecular hybridization to demonstrate viral genome in the malignant cells and in other non-transformed cells of the alimentary canal.

We have found that the method of keeping cattle in these areas is that one or 2 milk cows are allowed to graze with the larger numbers of the more common beef animals. This is a relatively unusual situation and means that milk is being ingested locally which will certainly contain carcinogen. An investigation is in progress to study any possible relationship with alimentary carcinoma in the human.